# Black Rainbow: How Words Healed Me – My Journey Through Depression

**DOI:** 10.1192/pb.bp.114.048397

**Published:** 2015-06

**Authors:** Rebecca J. Lawrence

**Figure:**
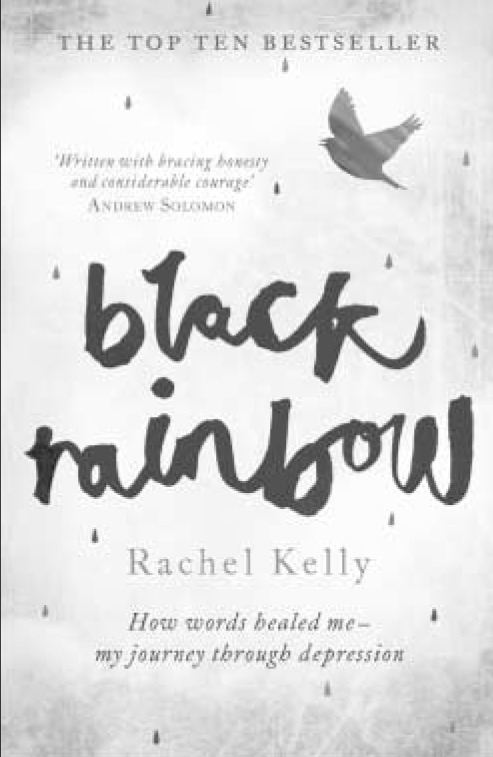


*Black Rainbow* is Rachel Kelly’s story of depression and recovery. It is an eloquent description of her experience of two severe depressive episodes, both with marked anxiety symptoms, and with a strong emphasis on the ‘striking physicality of the illness’.

During her first episode, she focuses on the biological nature of her illness, becoming frighteningly dependent on her husband and mother and an attentive psychiatrist, and obsessively preoccupied with her medication. Although this persists in the second episode, she develops a wider interest in factors that may have contributed to her illness, and seeks lifestyle changes and therapy to reduce her vulnerability. She recognises in particular her traits of sensitivity and perfectionism, and the difficulties inherent in combining motherhood with a high-achieving career.

Kelly gains much solace from words, including poetry and prayer, during her prolonged recoveries. Her familiarity with poetry from childhood may underlie this and her accounts of her life when depressed describe a return to a childlike state, where she is cared for by her devoted husband and mother. Her own role as a mother is temporarily lost, something she reflects on later with a sense of shame and failure.

Although she does not spare herself, it must be acknowledged that her experiences are different from most, given her level of privilege. A full-time nanny cares for her children, her psychiatrist visits her at home every couple of days, and she gives up work without obvious financial pressure, assuming a prolonged sick role. Interestingly, she herself questions the value of this and explores the difficulty of needing to be seen as either fully ill or well, and the possibility, often denied, of secondary gain. But her recovery is allowed to be unusually gentle, with a gradual and vividly recounted reawakening of senses dulled by depression, something not always possible for those less fortunate.

More personally, having also experienced depression, I found this a beautiful book. I remain unconvinced that poetry can cure depression (Kelly does not claim this), but it can provide much needed comfort and sets it within the human experience. In W.H. Auden’s words from *Musée des Beaux Arts*, ‘About suffering they were never wrong, The Old Masters’.

